# Pyrite-Based Autotrophic Denitrifying Microorganisms Derived from Paddy Soils: Effects of Organic Co-Substrate Addition

**DOI:** 10.3390/ijerph191811763

**Published:** 2022-09-18

**Authors:** Baokun Xu, Xiaoxia Yang, Yalong Li, Kejun Yang, Yujiang Xiong, Niannian Yuan

**Affiliations:** 1 Agricultural Water Conservancy Department, Changjiang River Scientific Research Institute, Wuhan 430010, China; 2 State Key Laboratory of Water Resources and Hydropower Engineering Science, Wuhan University, Wuhan 430072, China; 3 Key Laboratory of River Regulation and Flood Control of Ministry of Water Resources, Changjiang River Scientific Research Institute, Wuhan 430010, China; 4 Chongqing Water Resources Bureau, Chongqing 401147, China; 5 School of Law, Zhongnan University of Economics and Law, Wuhan 430073, China; 6 Agricultural and Rural Department of Hubei Province, Wuhan 430070, China

**Keywords:** pyrite, autotrophic denitrification, organic co-substrate, microbial community

## Abstract

The presence of organic co-substrate in groundwater and soils is inevitable, and much remains to be learned about the roles of organic co-substrates during pyrite-based denitrification. Herein, an organic co-substrate (acetate) was added to a pyrite-based denitrification system, and the impact of the organic co-substrate on the performance and bacterial community of pyrite-based denitrification processes was evaluated. The addition of organic co-substrate at concentrations higher than 48 mg L^−1^ inhibited pyrite-based autotrophic denitrification, as no sulfate was produced in treatments with high organic co-substrate addition. In contrast, both competition and promotion effects on pyrite-based autotrophic denitrification occurred with organic co-substrate addition at concentrations of 24 and 48 mg L^−1^. The subsequent validation experiments suggested that competition had a greater influence than promotion when organic co-substrate was added, even at a low concentration. *Thiobacillus*, a common chemolithoautotrophic sulfur-oxidizing denitrifier, dominated the system with a relative abundance of 13.04% when pyrite served as the sole electron donor. With the addition of organic co-substrate, *Pseudomonas* became the dominant genus, with 60.82%, 61.34%, 70.37%, 73.44%, and 35.46% abundance at organic matter concentrations of 24, 48, 120, 240, and 480 mg L^−1^, respectively. These findings provide an important theoretical basis for the cultivation of pyrite-based autotrophic denitrifying microorganisms for nitrate removal in soils and groundwater.

## 1. Introduction

Nitrate nitrogen (NO_3_^−^-N) has become one of the most ubiquitous contaminants in groundwater owing to the excessive use of fertilizers, landfills, discharge from domestic and industrial wastes, and atmospheric deposition [1]. The remediation of nitrate-contaminated groundwater has become the focus of attention and research in recent years [2], and biological denitrification has gradually come to be regarded as a reliable and economic method for the treatment of nitrate-contaminated groundwater [3]. Most studies in this field have focused on heterotrophic denitrifying bacteria that require organic carbon compounds as electron donors and carbon sources [4]. Woodchips, starch, wheat straw, and synthetic polymers are readily degradable organic substrates that have been used for the denitrification of groundwater [2]. Autotrophic denitrifying bacteria require inorganic compounds as electron donors and carbon dioxide (CO₂) or bicarbonate as carbon sources. Among the inorganic compounds studied, the use of pyrite as the electron donor for autotrophic denitrification has recently gained increasing attention [5,6,7].

The microbial oxidation of pyrite by *Thiobacillus denitrificans* has been reported to play an important role in the natural attenuation of nitrate-contaminated groundwater [8,9]. Recently, pyrite-based autotrophic denitrification was used to treat nitrate-contaminated groundwater. Pyrite minerals can be utilized by *T. denitrificans* as the single electron donor for denitrification [10], and the addition of pyrite to groundwater and sediments collected from a nitrate-contaminated aquifer can activate indigenous denitrifying microorganisms and stimulate denitrification even without *T. denitrificans* inoculation [11]. For synthetic groundwater, pyrite-based autotrophic denitrification exhibits a considerable nitrate removal rate, low sulfate production, and a stable pH [5]. An integrated two-stage soil infiltration bioreactor incorporated with pyrite-based (mixotrophic) denitrification has been designed for domestic wastewater treatment, and has been demonstrated to effectively remove chemical oxygen demand (COD), total phosphorus, and ammonium [12]. Previous research has evaluated the performance of particulate pyrite-based autotrophic denitrification and sulfur-oxidizing denitrification in soil infiltration bioreactors and continuous flow systems [6,13]. The application of particulate pyrite-based autotrophic denitrification in brackish aquaculture wastewater treatment and low-carbon source stormwater treatment has been assessed [14,15], and the efficiency of pyrite serving as both an electron donor for denitrification and a phosphorus adsorbent has also been investigated [15,16]. However, the nitrate removal performance of pyrite-based autotrophic denitrification is not ideal in most cases. It is therefore particularly important to improve the autotrophic denitrification performance.

The effect of organic substrate addition on the performance of a particulate pyrite-based autotrophic denitrification process has been evaluated in up-flow packed bed bioreactors, and organic carbon addition has been found to improve particulate pyrite-based autotrophic denitrification performance, which is attributed to the promotion of mixotrophic metabolism [6]. In a previous study, a combined heterotrophic and pyrite-based ferrous autotrophic system was proposed to treat low-C/N ratio wastewater. The mixotrophic system was demonstrated to have a greater abundance of the *narG* gene, while the abundance of the *nirS* gene was lower (*p* < 0.05), possibly leading to nitrite accumulation [17]. The addition of pyrite significantly promoted total nitrogen removal, with an efficiency higher than 27.05% under low C/N ratio conditions, indicating that mixotrophic denitrification was achieved in a vertical-flow constructed wetland [18]. The performance of nitrogen and phosphorus removal, as well as the secondary pollution produced by a bioretention system under heterotrophic (corncob-amended), autotrophic (pyrite-amended), and mixotrophic (corncob-and-pyrite-amended) conditions were compared, and it was found that corncob-and-pyrite-layered bioretention could maintain a low COD effluent concentration with high stability and efficiency in treating dissolved nutrients [19]. The denitrification performance of a novel mixotrophic system using a pyrite and biodegradable polymer composite (PLA/PHBV/rice hulls) as an electron donor was investigated, and the average nitrate removal rate (16.3–40.6 mg-N/L/d) in the mixotrophic system was 37% higher than the combined rate in the single heterotrophic and autotrophic system used for comparison [20]. The presence of organic carbon in wastewater, rainwater, groundwater, and soils is inevitable, and the influence of organic carbon on pyrite-based denitrification should be considered. Even without the addition of an exogenous organic carbon source, the dead and lysed cells of autotrophic bacteria may act as a carbon source for heterotrophic bacteria [21], and the addition of pyrite to groundwater and sediment can stimulate both autotrophic and heterotrophic denitrifying bacteria [11].

Although a higher probability of autotrophic denitrification was indicated in a pyrite-based denitrification column, the positive correlation between NO_3_^−^-N removal efficiency and dissolved organic carbon removal [7] found in a previous study suggested that the role of organic carbon in denitrification should be further explored. All neutrophilic microbial-driven nitrate-dependent iron oxidation bacterial strains isolated to date are mixotrophic, requiring an organic co-substrate for growth [22,23]. However, little is known about why mixotrophic denitrification is superior to pyrite-based autotrophic denitrification. It is unknown whether organic co-substrates promote the growth of pyrite-based autotrophic denitrifying bacteria. The main purpose of this study was to enhance the understanding of the role of organic co-substrates on the growth of pyrite-based autotrophic denitrifying bacteria. The specific objectives were to: (1) investigate the effect of organic co-substrate (acetate) addition on the performance of the denitrification system; (2) explore the functional bacterial populations using high-throughput sequencing technology with the addition of organic co-substrates at different concentrations; and (3) compare the performance of pyrite-based denitrification with inoculum from denitrification systems with the addition of organic co-substrates at different concentrations. The results will provide deeper insight into the microbial responses to the addition of organic co-substrates during pyrite-based autotrophic denitrification.

## 2. Materials and Methods

### 2.1. Preparation of Materials

Particulate pyrite (0.15–0.25 mm) was purchased from Luanchuan Hengkai Metallurgical Materials Sales Co., Ltd. (Luoyang, China). To remove sulfate from the pyrite surface, the particulate pyrite was rinsed with both tap and ultra-pure water prior to the experiment, then dried at 105 °C in a vacuum-drying oven for 4 h, cooled, and stored in the dark for later use. The particulate pyrite contained 47.09% (*w*/*w*) Fe, 39.96% S, 10.40% O, 0.969% Si, and 0.279% Al [7].

Paddy soils were collected from the upper soil layer (0–20 cm) of a local farm in Chongzuo, Guangxi Zhuang Autonomous Region, China (22°55′ N, 106°44′ E), which has a typical subtropical monsoon climate and a mean annual rainfall of 1362 mm. The soil samples were air-dried, passed through a sieve to remove particles with diameters >1 mm, and homogenized. The physicochemical properties of the soil samples are described in a previous report [24]. Synthetic groundwater A was used in experiment A, which investigated the addition of an organic co-substrate (acetate) on the performance of the denitrification system. Synthetic groundwater A was prepared by adding KNO_3_ to deionized water and contained 1010 mg L^−1^ KNO_3_, 680 mg L^−1^ KH_2_PO_4_, and 840 mg L^−1^ NaHCO_3_. The initial nitrate concentration in this synthetic groundwater was 140 mg-N L^−1^. The concentrations of organic co-substrate (acetate) added in each treatment are shown in Table 1. Synthetic groundwater B was used in experiment B, which compared the performance of pyrite-based denitrification with inoculum from different organic co-substrate addition denitrification systems. Synthetic groundwater B was prepared by adding KNO_3_ to deionized water and contained 361 mg L^−1^ KNO_3_, 44 mg L^−1^ KH_2_PO_4_, and 400 mg L^−1^ NaHCO_3_. The initial nitrate concentration in this synthetic groundwater was 50 mg-N L^−1^.

Air-dried soil samples were activated at 60% soil moisture content at 30 °C for 3 days. The activated soils were used as the microbial source in experiment A. The suspension after the reaction of experiment A was cleaned with sterilized normal saline three times to remove the residual organic matter, and then the cleaned suspension served as the microbial source in experiment B.

### 2.2. Experimental Procedure

Batch incubations were used to determine the influence of the organic co-substrate (acetate) addition on the performance of the denitrification system. During experiment A, pyrite-based autotrophic denitrifying microorganisms derived from paddy soils were cultivated with the addition of different concentrations of co-substrate. All the denitrification experiments using particulate pyrite as electron donor were conducted in 500-mL anaerobic bottles, with the experiments repeated in triplicate. For experiment A, the anaerobic bottles were filled with 40 g of dried, washed pyrite and 400 mL of the synthetic groundwater A with different concentrations of acetate. The bottles were then sterilized at 121 °C for 30 min. The six parallel treatments, denoted as A0, A24, A48, A120, A240, and A480, were cultured at acetate concentrations of 0, 24, 48, 120, 240, and 480 mg-C L^−1^, respectively. The bottles were then inoculated with 0.4 g of the prepared activated soils. Un-inoculated bottles without the addition of organic co-substrates were used as controls. The bottles were evacuated with a vacuum pump for 10 min and then flushed with ultrahigh purity helium gas (99.999%) for 5 min. This procedure was repeated three times. All flasks were cultivated at 30 °C for 20 days. When nitrate or acetate was consumed, it was added to the initial concentration. After 20 days, the inoculated anaerobic bottles were removed from the cultivation chamber and left to stand at room temperature for 30 min, and 200 mL of supernatant was filtered through a 0.45-μm membrane filter and then stored at −4 °C for microbial community analysis.

For experiment B, the anaerobic bottles were filled with 40 g of dried, washed pyrite and 400 mL of synthetic groundwater B with the same composition, and sterilized at 121 °C for 30 min. The aim of experiment B was to validate the competition or promotion role of heterotrophic denitrification in the pyrite-based autotrophic denitrification conducted in experiment A. The six parallel treatments, denoted as B0, B24, B48, B120, B240, and B480, were inoculated with 10 mL of the cleaned suspension from corresponding treatments in experiment A (Table 2). The control treatment was inoculated with physiological saline. The bottles were evacuated with a vacuum pump for 10 min and then flushed with ultrahigh purity helium gas (99.999%) for 5 min. This procedure was repeated three times. Bottles inoculated with sterilized normal saline were used as controls. All flasks were cultivated at 30 °C for 20 days.

### 2.3. Analysis

A 10-mL sample was collected from each bottle once every 48 h, and the pH of the sample was measured immediately after collection using a Portable Water Quality Analyzer (HQ40d, Hach, Loveland, CO, USA) with a pH electrode. Samples were filtered through a 0.45-μm membrane filter. The acetate, NO_3_^−^-N, NO_2_^−^-N, and SO_4_^2−^-S concentrations in the filtrate were measured using ion chromatography (883 Compact IC pro system, Metrohm AG, Herisau, Switzerland) with a Metrosep A Supp 5 separation column. Then, 0.5 mL of each sample was treated with 9.5 mL of H_2_O_2_ (3%, *v*/*v*) to oxidize the sulfite and thiosulfate in the samples to sulfate, and the total sulfate in the oxidized sample was measured using ion chromatography (883 Compact IC pro system, Metrohm AG, Herisau Switzerland) with a Metrosep A Supp 5 separation column. The oxidized sulfite and thiosulfate were defined as the difference between the total sulfate concentration and the sulfate concentration. Ammonia nitrogen (NH_4_^+^-N) was measured with the salicylic acid spectrophotometry method using a spectrophotometer (UV2700, Shimadzu, Kyoto, Japan). The total iron (Fe_Total_) and ferrous iron (Fe^2+^) were measured based on 1, 10-phenanthroline spectrophotometry [7].

### 2.4. DNA Extraction and Illumina MiSeq Sequencing

Biomass samples were collected from the water samples at the end of experiment A to perform a comprehensive analysis of the microbial communities. An activated soil sample was marked Soil, and six supernatant samples collected from the different treatments were marked as A0, A24, A48, A120, A240, and A480. The whole DNA was extracted using the PowerSoil DNA extraction kit (MoBio Laboratories, Carlsbad, CA, USA) following the manufacturer’s instructions. Illumina MisSeq sequencing was conducted by the Shanghai MEIJI Gene Technology Co. Ltd. (Shanghai, China) using the MiSeq 2500 Sequencing System (Illumina, San Diego, CA, USA). The primers 338F (5′-ACTCCTACGGGAGGCAGCAG-3′) and 806R (5′-GGACTACHVGGGTWTCTAAT-3′) were used for the amplification of the V3–4 sequences. Statistics and bioinformatics analyses were conducted following previous procedures [25].

## 3. Results and Discussion

### 3.1. Effect of Organic Co-Substrate Addition on Denitrification Performance

**Figure 1:**
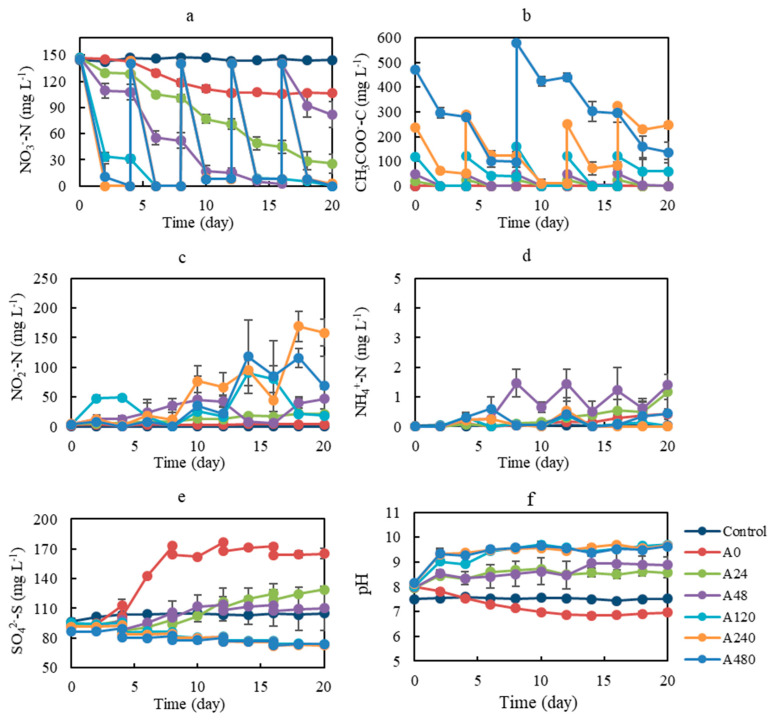
Changes in aqueous species concentrations during incubation experiment A (the cultivation of pyrite-based autotrophic denitrifying microorganisms derived from paddy soils with the addition of different concentrations of co-substrate). (**a**) Nitrate, (**b**) acetate, (**c**) nitrite, (**d**) ammonia nitrogen, (**e**) sulfate concentration, and (**f**) pH variation with different concentrations of co-substrate addition (un-inoculated bottles without the addition of organic co-substrates were used as controls. Treatments A0, A24, A48, A120, A240, and A480 were cultured with acetate addition at the concentrations of 0, 24, 48, 120, 240, and 480 mg-C L^−1^, respectively).

As shown in Figure 1, the addition of organic matter led to different denitrification performances in different treatments. During experiment A, the effects of high-concentration organic matter on the denitrification performance were estimated. Substrates (nitrate and acetate) were replenished to the initial concentration when they were close to depleted (less than 10% of initial concentration). In the control treatments, no activated soils or organic matter were added; as a result, the nitrate concentrations decreased very little. In treatments without added organic matter, the nitrate concentration decreased to 106.6 mg-N L^−1^ at the end of the experiments, and only 27.5% of the total nitrate was removed. In treatments where acetate was added at a concentration of 24 mg L^−1^, the nitrate concentration decreased to 25.6 mg-N L^−1^ at the end of the experiments, and 82.5% of the total nitrate was removed; acetate was replenished on days 4, 8, 12, and 16. In treatments where acetate was added at a concentration of 48 mg L^−1^, the nitrate concentration decreased to 2.7 mg-N L^−1^ and 98.2% of the total nitrate was removed on day 16; acetate was replenished on days 4, 8, 12, and 16, while nitrate was replenished on day 16. In treatments where acetate was added at a concentration of 120 mg L^−1^, nitrate was almost entirely consumed on day 6; acetate was replenished on days 4, 8, 12, and 16, while nitrate was replenished on days 8 and 16. In treatments where acetate was added at a concentration of 240 mg L^−1^, nitrate was almost entirely consumed and 100% of the total nitrate was removed on day 2; acetate was replenished on days 4, 12, and 16, while nitrate was replenished on day 4, 8, 12, and 16. In treatments where acetate was added at a concentration of 480 mg L^−1^, nitrate was almost entirely consumed and 100% of the total nitrate was removed on day 2; acetate was replenished on day 8, while nitrate was replenished on day 4, 8, 12, and 16. The nitrate reduction rate increased with the increase of acetate addition, which was in accordance with previous studies in which it was reported that organic carbon could improve particulate pyrite-based denitrification performance [6,7].

During experiment A, the accumulation of nitrite increased over time in each treatment, except for in the control treatments, in which no nitrite was produced. Among different treatments, the accumulation of nitrite increased with the increase of acetate addition, and thus the treatment without acetate addition exhibited the lowest amount of nitrite accumulation (Figure 1a). Ammonia nitrogen was produced in all the treatments, except for the control treatments. Even though its concentration appeared low (less than 2 mg L^−1^), the appearance of ammonia nitrogen implied a pathway of dissimilatory nitrate reduction to ammonium (DNRA). The relatively low concentration of ammonia nitrogen suggested that DNRA was not the main fate for nitrate removal.

During experiment A, the concentration of acetate decreased in the treatments with acetate addition. There are two fates for acetate consumption: the fraction of electrons transferred from donor to acceptor, and biomass [26]. The overall denitrification reactions using acetate can be represented as follows:

(1) $$0.097NO_{3}^{-}+0.125CH_{3}COO^{-}+0.097H^{+}\to \;0.037N_{2}+0.022C_{5}H_{7}O_{2}N+0.013CO_{2}+0.096H_{2}O+0.125HCO_{3}^{-}$$

Sulfate was produced during pyrite-based denitrification. As pyrite was the only electron donor, the amount of sulfate increased from 92.0 mg-S L^−1^ to 165.3 mg-S L^−1^ in treatments without organic matter addition. In treatments where acetate was added at a concentration of 24 or 48 mg L^−1^, less sulfate was produced, which could be attributed to the competitive or inhibitory role of acetate. Taking into account the simultaneous occurrence of nitrate decrease, acetate decrease, and sulfate increase, the competition between acetate and pyrite as electron donors was the reason for the lower sulfate. In treatments where acetate was added at a concentration of 24 or 48 mg L^−1^, acetate was the main electron donor; however, when acetate was insufficient, pyrite also provided some electrons for denitrification.

In treatments where acetate was added at a concentration higher than 120 mg L^−1^, only heterotrophic denitrification occurred for sufficient sodium acetate, and thus, no sulfate production was observed. According to previous studies, acetate plays a dual role in heterotrophic denitrification processes. First, acetate serves as the electron donor in the dissimilatory nitrate reduction process. Secondly, acetate serves as the carbon source for cell synthesis, and the typical value for the fraction of acetate that serves as an electron donor has been found to be 37.5% [26]. According to reaction Equation (1), when the concentration of nitrate was 140 mg L^−1^, the theoretical concentration of acetate needed was 312.5 g L^−1^. However, for the treatments where nitrate could be completely removed without the production of sulfate, the total concentration of acetate added was only 240 mg L^−1^. This discrepancy might be attributed to the addition of soil as the source of bacteria. Although only a small amount of soil was added, some organic matter would inevitably be introduced. Furthermore, the fraction of acetate that serves as an electron donor might be higher than 37.5%.

**Figure 2:**
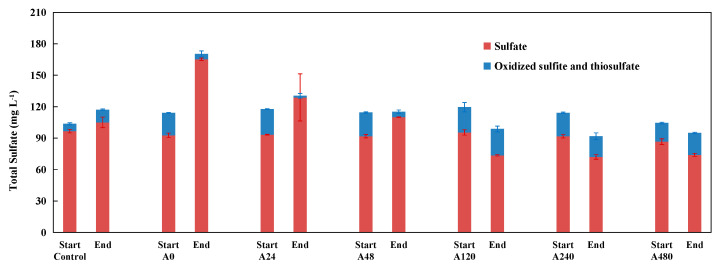
Changes of total sulfate (sulfate, oxidized sulfite, and thiosulfate) between the start and the end of the incubation experiment A (the cultivation of pyrite-based autotrophic denitrifying microorganisms derived from paddy soils with the addition of different concentrations of co-substrate).

In addition to sulfate, reduced sulfate (sulfite and thiosulfate) was also detected and remained stable before and after the reaction in treatments where acetate was added at concentrations of 120, 240, and 480 mg L^−1^ (Figure 2). This further demonstrated that no autotrophic denitrification occurred in these treatments, and suggested that the addition of organic matter at a high concentration may inhibit the pyrite-driven process of autotrophic denitrification. Reduced sulfate declined rapidly in the treatments without acetate addition, and the addition of acetate at low concentrations mitigated the decreasing trend of reduced sulfate. The decrease of reduced sulfate in treatments with acetate addition at 48 mg L^−1^ was more rapid than that in the treatment with acetate addition at 24 mg L^−1^, indicating that the addition of organic matter at a low concentration did not inhibit the pyrite-driven process of autotrophic denitrification, but may have instead promoted pyrite-driven autotrophic denitrification. Moreover, the addition of an organic co-substrate might compete with pyrite for electron acceptors even at a low concentration. Whether such competition or promotion mattered needs further confirmation.

During the experiment, the changes in pH varied among different treatments (Figure 1f). In the treatments without organic matter addition, the pH gradually decreased with time from ~8.3 to ~6.9. With the addition of acetate at concentrations of 24, 48, 120, 240, and 480 mg-C L^−1^, the pH increased to 8.6, 8.9, 9.7, 9.7, and 9.6, respectively, after 20 days of culture. The pH increased with time and the increment was larger in the treatments with the addition of more acetate. As has been reported, a high pH may benefit the accumulation of nitrite during denitrification [27,28,29]. Glass and Silverstein [27] observed that denitrification was significantly inhibited at pH 6.5, and that the nitrite accumulation rate increased significantly as the pH increased from 7.5 to 9.0. In a previous experiment based on wastewater treatment plant influent, partial denitrification was achieved under long-term high pH conditions, and nitrite accumulation rates increased with increasing influent pH from 5.0 to 9.0 [29]. Most of the reported heterotrophic nitrification-aerobic denitrification bacteria prefer neutral or alkaline conditions, while nitrification and denitrification are strongly inhibited at pH values below 6 [28,30,31,32]. In contrast, the acid production that occurs during denitrification using sulfur compounds as electron donors results in a pH drop [33]. The optimal pH condition was found to be 6.77 in an H_2_S-based denitrification system [34], while the optimum pH for sulfur-oxidizing bacteria was found to be 6.0–8.0 [6]. In the present study, the alkaline pH in the treatments with more acetate addition was suitable for heterotrophic denitrification, while the acid pH in the treatments with no acetate addition was suitable for sulfur-driven autotrophic denitrification.

### 3.2. Microbial Community Analyses with the Addition of Different Concentrations of Organic Co-Substrate in the Pyrite-Based Autotrophic Denitrification System

Through high-throughput sequencing, 475,777 effective sequences were obtained using 16S rRNA Illumina MiSeq sequencing. Table 3 shows the indexes of microbial diversity at a similarity of 97% in the seven samples (activated soil, denoted as Soil, and water samples with the addition of different concentrations of organic co-substrate after 20 days of incubation, denoted as A0, A24, A48, A120, A240, and A480).

The coverage index of the seven samples was high (>0.99), indicating a sufficient sequencing depth for community composition analysis. The operational taxonomic units (OTUs) of the seven samples differed, which indicated that organic co-substrate addition greatly influenced the bacterial abundances in this pyrite-based autotrophic denitrification system. The highest bacterial abundances were obtained in soil samples, while the lowest bacterial abundances were obtained in water samples with high co-substrate addition. Among the six water samples, the bacterial abundances decreased with the concentration of co-substrate addition. The Chao1 and Ace indexes represented the richness in the microbial community structure, while Shannon and Simpson indexes reflected the statistical diversity and evenness [35]. As shown in Table 3, the Ace, Chao1, and Shannon indexes of the seven samples had a similar trend to that of the OTUs, while the Simpson index exhibited the opposite trend, which also showed that the soil sample had the highest diversity and microbial richness, and that the water samples with high co-substrate addition had the lowest. The microbial diversity of the pyrite-based autotrophic denitrification system showed a decreasing trend with the increase of the concentration of co-substrate addition, implying that the continuous addition of organic matter might reduce the relative abundances of some microorganisms (such as autotrophic denitrification microorganisms) in soils, which was consistent with the decreasing sulfate production.

**Figure 3:**
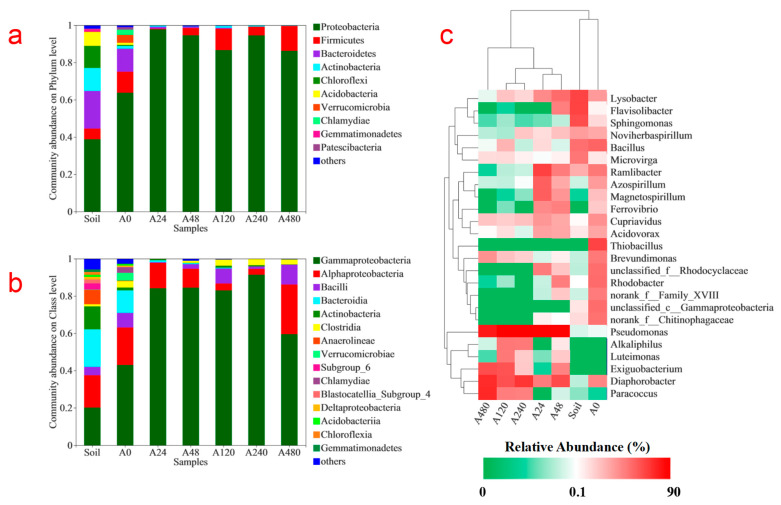
Microbial structure of activated soil and water samples at the phylum level (**a**), class level (**b**), and genus level (**c**). The heatmap illustrates the relative abundances (%).

The phylogenetic classification of obtained microbial sequences from bacterial communities of the soil samples and six water samples was performed, and the sequences were assigned to different taxonomic levels (phylum, class, and genus). On the basis of taxonomic studies, a total of 25 bacterial phyla were detected. As presented in Figure 3a, the most dominant phyla (>3% abundance in at least one sample) in the soil samples were Proteobacteria (38.8%), Bacteroidetes (20.2%), Actinobacteria (12.3%), Chloroflexi (11.9%), Acidobacteria (7.4%), and Firmicutes (5.7%). Compared with soil samples, the microbial community of the pyrite-based autotrophic denitrification system changed significantly at the phylum level under different concentrations of co-substrate addition. For the A0 treatment, the relative abundances of Proteobacteria (63.8%), Firmicutes (11.2%), and *Verrucomicrobia* (4.2%) increased significantly, while the relative abundances of other phyla decreased by different degrees. For the A24 treatment, Proteobacteria was the only dominant phylum, and accounted for 97.8% of all microbial species. For the A48, A120, A240, and A480 treatments, Proteobacteria was also the most dominant phylum, and accounted for 94.6%, 86.7%, 94.6%, and 86.3% of all microbial species, respectively, while the relative abundances of Firmicutes, the second most dominant phylum, were 3.9%, 11.4%, 4.7%, and 13.3%, respectively.

The microbial members of Acidobacteria are widely distributed in all types of soils [36]. These microorganisms often grow slowly in poor soil, and their relative abundance is negatively correlated with soil available organic matter [37], and is significantly affected by pH [38,39]. The *narB*, *narG*, *nirA*, *norB*, and *norC* genes were found in a genome analysis of the microorganisms in Acidobacteria, indicating that the microorganisms in Acidobacteria can participate in denitrification [40]. The microorganisms of Acidobacteria also have the function of using Fe(II) and other reducing inorganic substances to drive denitrification under anaerobic or hypoxic conditions [40]. Bacteroidetes can secrete different carbohydrate-active enzymes that are targeted for polysaccharides in soils, and are thought to be used for the degradation of complex organic compounds [41]. Many Bacteroidetes microorganisms are also considered denitrification microorganisms. Bacteroidetes are often found in constructed wetlands, and the addition of zero-valent iron has been demonstrated to promote the increase of the relative abundance of Bacteroidetes, thus improving the relative abundance of denitrification microorganisms [42,43]. Actinobacteria were found in denitrification systems in previous research [25,44,45], and showed good nitrate removal abilities and chromium reduction efficiency under neutral conditions for higher denitrification [46]. Proteobacteria and Firmicutes were found to be the dominant phyla in a laboratory-scale combined bioelectrochemical and sulfur autotrophic denitrification system and an expanded granular sludge bed reactor for the simultaneous biological removal of sulfate, nitrate, and lactate [47,48]. Proteobacteria contains many types of metabolic bacteria, and is responsible for the removal of COD and nitrogen, making Proteobacteria the most abundant phylum in sewage treatment and sulfur autotrophic denitrification processes [35,49,50,51]. In a novel three-dimensional bioelectrochemical denitrification system, Firmicutes was found to be the dominant community during the denitrification process, and worked best under a pH 7.0–8.0 environment, while Proteobacteria and Actinobacteria preferred acid or alkaline environments in the three-dimensional bioelectrochemical denitrification system [25]. Proteobacteria and Chloroflexi were the major phyla in a pilot-scale sulfur-limestone autotrophic denitrification biofilter at low temperatures [52]. Microorganisms belonging to the Chloroflexi phylum are often found in autotrophic nitrogen removal systems, which are conducive to the degradation of dead microorganisms and the agglomeration of granular sludge [53].

In a previous study, it was found that Firmicutes, Proteobacteria, and Bacteroidetes harbored all four nitrate reductive genes (*narG*, *nir*, *nosZ*, and *nor*), which are responsible for nitrate, nitrite, NO, and N_2_O reduction, respectively [54]. The denitrification performance of the pyrite-based autotrophic denitrification systems improved with high relative abundances of Firmicutes, Proteobacteria, and Bacteroidetes, suggesting that Firmicutes, Proteobacteria, and Bacteroidetes play important roles in pyrite-based autotrophic denitrification systems without the addition of organic co-substrates. Moreover, the high relative abundance of Verrucomicrobia implied that this phylum might play an important role in pyrite-based autotrophic denitrification systems without organic co-substrate addition. The proportions of Proteobacteria and Firmicutes in the pyrite-based autotrophic denitrification systems with organic co-substrate addition increased, while the proportion of Bacteroidetes significantly decreased, indicating that Bacteroidetes only played a role in the pyrite-based autotrophic denitrification system, while Proteobacteria and Firmicutes played important roles in both the pyrite-based autotrophic denitrification and heterotrophic denitrification systems.

Based on further taxonomic investigation, a total of 66 bacterial classes were detected in the soil sample and the six water samples at the class level. As displayed in Figure 3b, the most dominant classes (>3% abundance in at least one sample) in the soil samples were Gammaproteobacteria (20.1%), Alphaproteobacteria (17.3%), Bacilli (4.5%), Bacteroidia (20.1%), Actinobacteria (12.3%), Anaerolineae (7.7%), and Subgroup_6 (3.4%). For the A0 treatment, the most dominant classes (>3% abundance in at least one sample) were Gammaproteobacteria (43.0%), Alphaproteobacteria (20.1%), Bacilli (7.8%), Bacteroidia (12.1%), Clostridia (3.4%), and Verrucomicrobiae (4.2%). The relative abundances of Gammaproteobacteria, Alphaproteobacteria, Bacilli, Clostridia, and Verrucomicrobiae increased significantly, while the relative abundances of other classes decreased to different degrees. These increased microbial classes (Gammaproteobacteria, Alphaproteobacteria, Bacilli, Clostridia, and Verrucomicrobiae) might play an important role in pyrite-driven autotrophic denitrification. For the A24 treatment, Gammaproteobacteria and Alphaproteobacteria were the main microbial classes, accounting for 84.2% and 13.6%, respectively. The relative abundance of Gammaproteobacteria increased, while Alphaproteobacteria decreased in the A24 treatment due to the continuous replenishment of organic co-substrates. These findings suggest that the microorganisms that can participate in both pyrite-based denitrification and organic-driven heterotrophic denitrification may mainly belong to Gammaproteobacteria. For the A48, A120, A240, and A480 treatments, the most dominant class was Gammaproteobacteria, accounting for 84.5%, 83.0%, 91.4%, and 59.5% of all microbes, respectively, followed by Alphaproteobacteria, accounting for 3.9%, 11.4%, 4.7%, and 13.3%, respectively. Moreover, Bacilli and Clostridia were also dominant classes in the A120, A240, and A480 treatments.

Alphaproteobacteria have been reported to play an important role in bioelectrochemical denitrification [55]. In a novel three-dimensional bioelectrochemical denitrification system, Clostridia were the most important contributor to the highest nitrate removal under pH 7.0–8.0 conditions, followed by Bacilli, Gammaproteobacteria, and Alphaproteobacteria [25]. Additionally, the most abundant class at pH 9.0 was Bacilli, while Gammaproteobacteria were the most abundant class at pH 6.0, meaning that the acid environment was more suitable for Gammaproteobacteria in the bioelectrochemical denitrification system [25]. Most reported hydrogenotrophic denitrification bacteria belonged to the Alphaproteobacteria, Betaproteobacteria, and Gammaproteobacteria classes, and Betaproteobacteria were the dominant class in a hydrogen-oxidizing autotrophic denitrifying system [56]. Denitrification is also a common feature among members of the genus *Bacillus* [57]. Gammaproteobacteria (mainly *Pseudomonas*) and Epsilonproteobacteria (mainly *Arcobacter* and *Sulfurospirillum*) were found to increase with the increase of nitrate concentration in systems that removed both sulfates and nitrates [58]. Betaproteobacteria and Epsilonproteobacteria were the main denitrification bacteria in large sulfur/limestone autotrophic denitrification filters under low-temperature conditions [52]. Bacteroidia has been reported in a combined bioelectrochemical and sulfur autotrophic denitrification system, an anoxic–aerobic sequential batch reactor, and a mining clarifying pool [48,50,59,60], suggesting that some microorganisms in the Bacteroidia class might play a role in the denitrification process. Acidobacteria are a major microbial class in the phylum Acidobacteria. The relative abundance of Acidobacteriia is related to environmental pH [38,39,58], and this class has an ability to use Fe(II) and other reducing inorganic substances to drive denitrification under anaerobic or hypoxic conditions [40]. Previous studies have shown that microorganisms in the Anaerolineae class are involved in the heterotrophic denitrification process, and dead microorganisms during the autotrophic denitrification process can also be used as a carbon source for these heterotrophic denitrification processes [61,62,63]. The Verrucomicrobiae class was found in both a combined anaerobic/aerobic reactor and soil [64,65], suggesting that these microbes might be associated with activated sludge systems and denitrification processes.

In the present study, Gammaproteobacteria and Alphaproteobacteria were the main bacterial groups in all treatments, while Bacteroidia and Verrucomicrobiae were major bacterial groups only in the pyrite-based autotrophic denitrification system without the addition of an organic co-substrate. Based on the relative abundance changes of microorganisms at the class level and the findings of previous studies, Gammaproteobacteria, Alphaproteobacteria, Bacilli, Clostridia, and Verrucomicrobiae played major roles in the pyrite-based autotrophic denitrification process.

There were a total of 466 genera in the six water samples and in the sampled soil. The top 25 genera in all samples were selected and compared to determine their relative abundances and generate a heatmap. Generally, both the abundant genera and their relative abundances varied widely between samples. *Flavisolibacter* (12.77%), which belongs to the Bacteroidetes phylum and has been found to have a significant positive correlation with the number of denitrification functional genes in soil [66], was the most predominant genus within the soil sample, while the dominant genera shifted to *Thiobacillus* (13.04%), *Pseudomonas* (60.82%), *Pseudomonas* (61.34%), *Pseudomonas* (70.37%), *Pseudomonas* (73.44%), and *Pseudomonas* (35.46%) in the six water samples with different concentrations of organic co-substrates.

In soil samples, *Lysobacter* (12.36%), *Sphingomonas* (9.93%), *Bacillus* (4.17%), and *Microvirga* (3.55%) were also over 3%. *Lysobacter*, which belongs to the Proteobacteria phylum, was a possible facultative autotrophic denitrifier [67,68]. *Sphingomonas*, belonging to the Proteobacteria, has not only shown an excellent ability to degrade phenolic compounds and polyhydroxyalkanoates, but also has a high nitrogen removal capacity, making it a prospective heterotrophic nitrification-aerobic denitrification bacterium for the simultaneous removal of organic matter and nitrogen [69]. *Bacillus*, which belongs to the Firmicutes phylum, was found to be related to the nutrient removal efficiencies of *Bacillus* sp. using sodium acetate as a sole carbon source [70]. In addition, the heterotrophic nitrification, aerobic denitrification, and denitrifying phosphorous removal ability of *Bacillus* genera have been demonstrated [31,70]. *Microvirga* belong to the Proteobacteria phylum and are nitrogen-fixing bacteria that can increase nitrogen contents through N_2_ fixation [71]. The community structure distribution at the microbial genus level indicated that there was strong nitrification and denitrification potential for microorganisms in these soils.

In the A0 treatment, the most dominant genus was *Thiobacillus* (13.04%). *Thiobacillus* is the major autotrophic denitrifier reported in most sulfur-based autotrophic denitrification systems, which can use a wide range of reduced sulfur compounds, such as elemental sulfur, sulfide, and thiosulfate, as electron donors for nitrate reduction [72]. Therefore, the high relative abundance of *Thiobacillus* in the A0 treatment indicated the successful enrichment of the sulfur-based autotrophic denitrifying community from the paddy soils. In addition, the water samples also contained *Bacillus* (5.80%), *Rhodobacter* (4.92%), *unclassified_c__Gammaproteobacteria* (4.74%), *unclassified_f__Rhodocyclaceae* (4.67%), *norank_f__Chitinophagaceae* (4.27%), *Ramlibacter* (3.53%), *Brevundimonas* (3.48%), and *norank_f__Family_XVIII* (3.23%).

*Rhodobacter*, a genus belonging to Proteobacteria, is capable of aerobic denitrification [73]. *Unclassified_c__Gammaproteobacteria* from Gammaproteobacteria plays a vital role in the nitrogen removal process [74]. *Unclassified_f__Rhodocyclaceae* belongs to a denitrifying phosphorus-accumulating bacteria group [75]. *Norank_f__Chitinophagaceae* belongs to *Chitinophagales*, among which most microorganisms are aerobic or facultative anaerobic microorganisms [76]. *Flavisolibacter*, from the same family as *norank_f__Chitinophagaceae*, has been found to be significantly positively correlated with the number of denitrifying functional genes [66]. *Ramlibacter*, from Proteobacteria, is associated with nitrification [76,77]. The *Brevundimonas* species are ubiquitous in the environment, being one of few bacterial species with high survival rates under extremely harsh conditions [78]. *Brevundimonas denitrificans* sp. nov., a novel bacterium with potential denitrification ability, was previously isolated from deep seafloor sediment in Japan [79]. *norank_f__Family_XVIII* also accounted for a large proportion in the A0 treatment, which needs to be further investigated. In general, sulfur autotrophic denitrifying microorganisms were dominant. A small number of denitrifying microorganisms in the A0 treatment used organic matter or iron as electron donors.

In the treatments with organic matter addition at low and high concentrations, the denitrifying microorganisms were mainly *Pseudomonas* with a small number of other heterotrophic/autotrophic denitrifying microorganisms. In the A24 treatment, the most dominant bacterium was *Pseudomonas* (60.82%), a widely studied heterotrophic denitrifier [80,81]. In recent years, this bacterium has also been found to play a vital role in manganese autotrophic denitrification, iron autotrophic denitrification [81], heterotrophic/sulfur autotrophic denitrification [58], and bioelectrochemical denitrification [48]. Other dominant genera (relative abundance > 3%) were *Ramlibacter* (11.75%), *Magnetospirillum* (5.65%), and *Azospirillum* (5.47%). *Magnetospirillum* and *Azospirillum* are newly emerging dominant species detected in the A24 treatment. *Magnetospirillum* contains genes capable of compiling nitrate reductase, nitrite reductase, nitric oxide reductase, and nitrous oxide reductase, with complete denitrification capability [82]. *Azospirillum* has complete denitrification functional genes and can fix nitrogen [83,84]. In the A48 treatment, the dominant bacteria (relative abundance > 3%) were *Pseudomonas* (61.34%), *Diaphorobacter* (9.54%), and *Lysobacter* (4.09%). *Diaphorobacter* can use organic matter as an electron donor to reduce nitrate to nitrogen gas [85]. In addition, *Ferrovibrio*, a neutral anaerobic iron-oxidizing microorganism with denitrification function, was also found in the A24 and A48 treatments; its denitrification chain was interrupted and did not exhibit nitrite reduction function [86].

In the A120 treatment, *Pseudomonas* (70.37%) was the most dominant bacterium. Other dominant genera (relative abundance >3%) included *Diaphorobacter* (7.98%), *Exiguobacterium* (7.37%), and *Alkaliphilus* (3.01%). *Exiguobacterium* plays important roles in the sulfide denitrification system and sulfur autotrophic denitrification process [87]. *Alkaliphilus* microbes can grow with acetate as a carbon source under alkaline conditions [88]. In both the A240 and A480 treatments, *Pseudomonas* was the most dominant bacterium, similar to the A120 treatments. In general, the dominant denitrifying bacteria were heterotrophic denitrifying microorganisms in the A120, A240, and A480 treatments due to the excessive addition of organic matter. There were also many other heterotrophic/autotrophic denitrifying microorganisms, but their relative abundances differed from treatments with the continuous addition of low-concentration organic matter. The results were similar to those of a study on an integrated heterotrophic and autotrophic denitrification process for organic-limited polluted water, which showed that limited organic content could accelerate the nitrate removal rate [89].

In general, soil microbial communities are diverse and abundant in agricultural soil, which was demonstrated to be a potential source of autotrophic denitrification microorganisms for pyrite. In the denitrification system using pyrite as the sole electron donor, *Thiobacillus*, which could use sulfur as an electron donor, was the most dominant bacterium. The continuous replenishment of organic co-substrate, however, led to a different microorganism composition. In the treatments with the continuous replenishment of organic co-substrate at low or high concentrations, the denitrifying microorganisms were dominated by *Pseudomonas* in almost all cases, but other different heterotrophic/autotrophic denitrifying microorganisms were found among treatments with different concentrations of organic co-substrate.

### 3.3. Performance of Pyrite-Based Denitrification with Inoculum from Denitrification Systems with the Addition of Different Concentrations of Organic Co-Substrate

**Figure 4:**
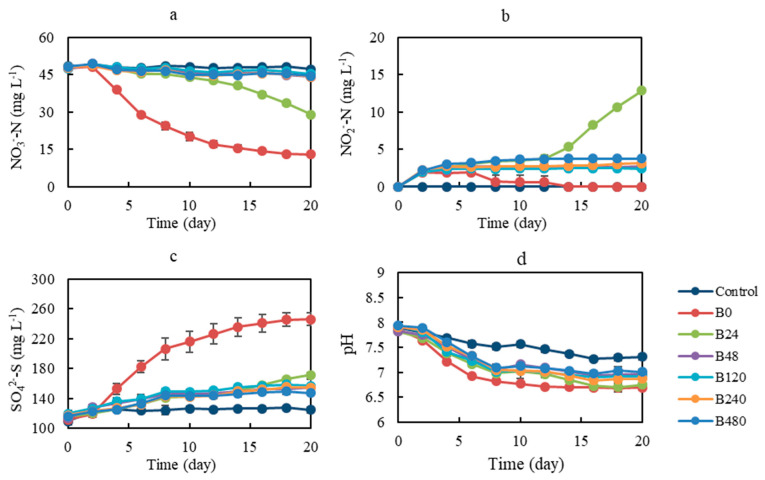
Changes in aqueous species concentrations during incubation experiment B (validation of the competition or promotion role of heterotrophic denitrification with pyrite-based autotrophic denitrification). (**a**) Nitrate, (**b**) nitrite, (**c**) sulfate concentration, and (**d**) pH variation with different microbial sources (the control treatments was inoculated with physiological saline, and experimental treatments inoculated with microbes from the A0, A24, A48, A120, A240, and A480 treatments were defined as B0, B24, B48, B120, B240, and B480, respectively).

For experiment B, six suspensions from experiment A were used as bacterial sources to further verify their autotrophic denitrification ability using pyrite as the sole electron donor. As shown in Figure 4, the concentration of nitrate remained almost unchanged in the control, indicating that nitrate could not be effectively chemically reduced by pyrite in the absence of a bacterial source. In the B0 treatment, the nitrate concentration decreased with the increase in incubation time, and about 72.8% of the total nitrate was removed at the end of the incubation experiment. In the B24 treatment, the nitrate concentration decreased with the increase of incubation time, but the nitrate removal efficiency was only 39.7% at the end of the incubation experiment. The nitrate concentration decreased only slightly during incubation, and the nitrate removal efficiency was only 6.8%, 4.5%, 7.8%, and 7.9% in B48, B120, B240, and B480 treatments, respectively.

During the whole experiment, no accumulation of nitrite was observed in the control, which demonstrated once again that no pyrite-based denitrification occurred in the control. In the B0 treatment, nitrite first increased and then decreased, and the maximum accumulation was 1.9 ± 0.1 mg L^−1^. In the B24 treatment, nitrite accumulated slowly to 3.8 ± 0.4 mg L^−1^ in the first 12 days, and from day 12 onward, the accumulation of nitrite increased sharply and reached a final concentration of 12.9 ± 0.2 mg L^−1^ at the end of the incubation. In the B48, B120, B240, and B480 treatments, nitrite accumulated slowly, and the maximum concentrations were 2.7 ± 0.6, 2.5 ± 0.3, 3.1 ± 0.8, and 3.8 ± 0.1 mg L^−1^, respectively.

The different nitrate removal efficiencies in experiment B were mainly due to different bacterial sources. The high nitrate removal efficiency in the B0 treatment indicated that suspensions from the A0 treatment (without organic co-substrate addition) in experiment A could be used as the bacteria source for pyrite-based autotrophic denitrification. Microbial community structure analysis revealed the dominant bacterial sources obtained were mainly *Thiobacillus* in the A0 treatment, which was consistent with the high nitrate removal efficiency in the B0 treatment. *Thiobacillus* can participate in pyrite-based denitrification [11]. Suspensions from the A24 treatment (with organic co-substrate addition at 24 mg L^−1^) in experiment A can be used as the bacteria source for pyrite-based autotrophic denitrification. However, the autotrophic denitrification capacity of the suspensions obtained from the A24 treatment was not as strong as that obtained from the A0 treatment. Based on the significantly lower nitrate removal efficiency and the greater accumulation of nitrite in the B24 treatment compared to that in the B0 treatment, it was concluded that the dominant bacteria in the A24 treatment consisted of heterotrophic denitrifying microorganisms, and that there were also microorganisms that could use pyrite to reduce nitrate to nitrite. This conclusion was supported by the microbial community structure analysis, which showed that *Pseudomonas* was the most dominant bacterial genus, and the relatively high microbial abundance of *Ferrovibrio*, whose denitrification chain was interrupted and did not exhibit a nitrite reduction function. No significant denitrification occurred in the B48, B120, B240, and B480 treatments, suggesting that the continuous supplementation of organic co-substrate weakened the autotrophic denitrification capacity of suspensions. Based on these findings in combination with the results of experiment A, it was concluded that the competition effect of organic co-substrate addition on pyrite-based autotrophic denitrification had a stronger influence than the promotion effect, even at a concentration of 24 mg L^−1^.

The detected ammonia nitrogen content was relatively low during the incubation experiments. In the B0 treatment, the ammonia nitrogen concentration decreased after first increasing to a maximum concentration of 0.2 ± 0.0 mg L^−1^. With the degradation of nitrate, ammonia accumulation gradually occurred, which might have been mainly due to the occurrence of DNRA or the ammonization of dead cells [90]. The subsequent decrease in ammonia nitrogen may have been caused by assimilation by microorganisms because ammonium is taken up preferentially by microorganisms for growth [2]. These results were consistent with reports on other denitrification systems using pyrite as an electron donor [6,7]. In the B24, B48, B120, B240, and B480 treatments, the concentration of ammonia nitrogen remained below 0.1 ± 0.1 mg L^−1^, although an increasing trend was observed.

**Figure 5:**
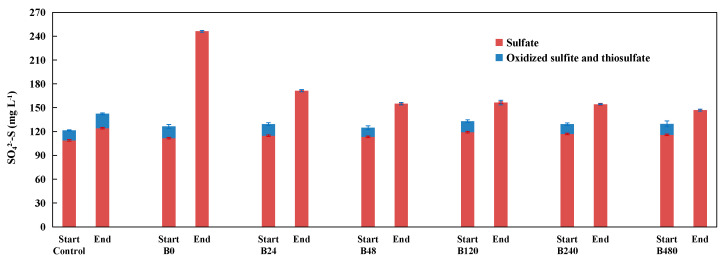
Changes of total sulfate (sulfate, oxidized sulfite, and thiosulfate) between the beginning and the end of incubation experiment B (validation of the competition or promotion role of heterotrophic denitrification with pyrite-based autotrophic denitrification).

As presented in Figure 5, in the control, the sulfate content only increased by 15.4 mg L^−1^ during incubation, and its increment was the lowest among all treatments. The increase of sulfate in the control was probably due to the chemical oxidation of pyrite by the initial entrained air. In addition to sulfate, increases in thiosulfate and sulfite were also found in the control. According to previous studies, sulfite can be utilized by *Sulfurimonas denitrificans* and may be an ideal intermediate product during denitrification [5,91]. It is speculated that biological denitrification processes may not occur when there is a lack of related microorganisms. In the B0 treatment, the sulfate content increased by 134.6 mg L^−1^, and its increment was the greatest among all treatments. The increase of sulfate in the B0 treatment was mostly due to the biological oxidation of pyrite by pyrite-based autotrophic denitrification. In the B24, B48, B120, B240, and B480 treatments, the increments of sulfate were 56.6 mg L^−1^, 41.6 mg L^−1^, 37.2 mg L^−1^, and 31.0 mg L^−1^, respectively, and the accumulation of sulfate in these treatments was lower than that in the B0 treatment owing to the weaker pyrite-based autotrophic denitrification in these treatments. In all treatments with inoculum, thiosulfate and sulfite quickly decreased to below the detection limit, suggesting that thiosulfate and sulfite may be preferentially utilized as electron donors by denitrifying microorganisms.

During the incubation experiments, both water-soluble total iron and Fe(II) were below the detection limit. These results were consistent with previous research [6,7]. Fe(II) in pyrite is believed to be involved in denitrification and produce Fe(OH)_3_ precipitation.

The pH showed a decreasing trend during the incubation experiments. The pH decreased the most slowly in the control, resulting in a decrement of 0.6. In the B0 treatment, the pH declined at its fastest pace and decreased by 1.2 during the incubation, followed by the B24 treatment, which resulted in a decrement of 1.1. Moreover, pH decreased by 0.9, 1.0, 1.0, and 0.9 in the B48, B120, B240, and B480 treatments during incubation. According to Equation (1), 0.48 mol of H^+^ was produced by reducing 1.00 mol of NO_3_^−^-N. However, the pH variation was insignificant, particularly when compared with sulfur-based denitrification [5]. The pH-buffering nature of the pyrite-based denitrification reaction was in accordance with previous research [5,7]. Thus, the pH decreased moderately during pyrite-based autotrophic denitrification, and the degree of pH decrease was positively correlated with the amount of nitrate removed.

### 3.4. Limitations and Outlook

Although this study has made contributions to research in related fields, it has some limitations. The first limitation is that the types of organic co-substrate used were not comprehensive enough. Low-molecular-weight organic carbon compounds can be considered as the key extracellular intermediaries when microbes anaerobically oxidize organic matter [92,93]. Simple sugars are the major types of low-molecular-weight organic carbon compounds, including acetate, propionate, glucose, and lactate, many of which can be readily metabolized by soil microorganisms [92,94]. Only acetate was used in the present study to investigate its role in pyrite-based autotrophic denitrifying microorganisms derived from paddy soils. Acetate cannot fully represent all the organic co-substrates in soils. The second limitation is the limited experimental setting. This study focused on the effects of acetate addition on the performance and bacterial community of pyrite-based denitrification processes. During the experiments, acetate was added several times at relatively high concentrations in order to obtain more remarkable results. However, the effects of low-concentration organic co-substrate addition on denitrification have not been studied. Metagenomic insights into the effects of acetate addition on pyrite-based denitrification processes might provide a deeper understanding of pyrite-based denitrification processes.

In the future, the roles of other types of organic co-substrates, such as glucose, propionate, and lactate, will be investigated. The effects of low-concentration organic co-substrate addition on pyrite-based denitrification processes will also be taken into account. Moreover, the bacterial community during pyrite-based denitrification processes will also be analyzed in greater depth. Further studies using 16S rRNA gene and metagenomic sequencing analysis will be conducted to reveal the bacterial diversity and community structures, as well as the relevant functional genes involved in nitrogen transformation and the pathways of nitrogen metabolism, which will lay a biological foundation for developing high-performance pyrite-based denitrification processes.

## 4. Conclusions

The addition of organic co-substrate had a great influence on pyrite-based autotrophic denitrification. First, the addition of an organic co-substrate at concentrations higher than 48 mg L^−1^ could inhibit the process of pyrite-based autotrophic denitrification. In addition, both competition and promotion effects on pyrite-based autotrophic denitrification occurred. It was found that competition had a greater influence than promotion when an organic co-substrate was added at low concentration. *Thiobacillus* dominated the denitrification system where pyrite served as the sole electron donor. However, with the addition of organic co-substrate *Pseudomonas* became the dominant genus.

## Figures and Tables

**Table 1:** Experiment A setup.

Treatment	Soil	Nitrate-N (mg L^−1^)	Acetate-C (mg L^−1^)	NaHCO_3_ (mg L^−1^)	Pyrite (g)
Control	0	140	0	840	30
A0	0.1%	140	0	840	30
A24	0.1%	140	24	840	30
A48	0.1%	140	48	840	30
A120	0.1%	140	120	840	30
A240	0.1%	140	240	840	30
A480	0.1%	140	480	840	30

**Table 2:** Experiment B setup.

Treatment	Microbial Source	Nitrate-N (mg L^−1^)	NaHCO_3_ (mg L^−1^)	Pyrite (g)
Control	Physiological saline	50	400	30
B0	A0	50	400	30
B24	A24	50	400	30
B48	A48	50	400	30
B120	A120	50	400	30
B240	A240	50	400	30
B480	A480	50	400	30

**Table 3:** Species richness and diversity estimators of microbial populations in soil and water samples.

Sample ID	Reads	OTUs ^2^	Ace	Chao1	Coverage	Shannon	Simpson
Soil ^1^	55,928	1180	1189.804	1185.194	0.999392	5.025932	0.028366
A0	52,525	378	386.8607	387.7308	0.999562	4.330376	0.024486
A24	64,104	133	188.8802	166	0.999485	1.714763	0.38743
A48	62,599	169	216.1763	238.4615	0.999313	1.886757	0.388363
A120	64,316	107	135.5286	141.3636	0.999565	1.310092	0.509113
A240	61,244	81	97.28298	96.3	0.999706	1.026962	0.564491
A480	54,153	82	104.8487	105.0769	0.999538	1.601675	0.250963

^1^ Activated soil is denoted as soil, and water samples with the addition of different concentrations of organic co-substrates after 20 days of incubation are denoted as A0, A24, A48, A120, A240, and A480. ^2^ OTUs: operational taxonomic units.

## Data Availability

Data that support the findings of the study are available from the corresponding author upon reasonable request.
